# The hepatic extramedullary hematopoiesis during experimental murine *Schistosomiasis mansoni*

**DOI:** 10.3389/fimmu.2022.955034

**Published:** 2022-08-25

**Authors:** Juliane Siqueira Francisco, Marcia Andrea Barge Loução Terra, Gabriel Couto Thurler Klein, Barbara Cristina Euzebio Pereira Dias de Oliveira, Marcelo Pelajo-Machado

**Affiliations:** ^1^ Laboratory of Pathology, Instituto Oswaldo Cruz, Fiocruz, Rio de Janeiro, Brazil; ^2^ Brazilian National Institute of Science and Technology on Neuroimmunomodulation, Oswaldo Cruz Institute, Oswaldo Cruz Foundation, Rio de Janeiro, Brazil

**Keywords:** schistosomiasis, granuloma, hematopoietic environment, angiogenesis, extramedullary hematopoiesis

## Abstract

Many years ago, our research group has demonstrated extramedullary hematopoiesis in the peripheral zone of murine hepatic schistosomal granulomas. In the present study, we revisit this phenomenon using new technical and conceptual approaches. Therefore, newborn mice were percutaneously infected by *Schistosoma mansoni* cercariae and euthanized between 35- and 60-days post infection. Liver samples were submitted to histopathology and immunohistochemical analyses. Cells under mitosis and/or expressing Ki67 demonstrated the proliferation of hematopoietic cells both around the parasite’s eggs trapped in the liver and around hepatic vessels. After 50 days post infection, proliferating cells at different levels on differentiation were located preferentially in the peripheral zone of the granulomas, around the vessels and inside the sinusoids. The presence of acidic and sulfated glycoconjugates, reticular fibers and the absence of fibronectin characterized the microenvironment for attraction and maintenance of hematopoiesis. Some neutrophils secreted MMP9 from the earliest points of infection, indicating degradation of the extracellular matrix in regions of histolysis and a possible chemoattraction of hematopoietic stem cells to the liver. Fall-3+ cells and Sca-1+ cells indicated that early hematopoietic progenitors could be mobilized to the liver. Groups of vWF+ megakaryocytes suggest chemoattraction of these cells and/or migration, proliferation, and differentiation of very immature progenitors to this organ. The increase of blood vessels and extramedullary hematopoiesis in this environment, where markers of immature hematopoietic and endothelial cells have been identified, points to the possibility of the presence of progenitors for endothelial and hematopoietic cells in the liver during the infection. There is also the possibility of concomitant migration of more differentiated hematopoietic progenitors, that proliferate and differentiate in the liver, and the occurrence of angiogenesis caused by inflammation or release of ovular antigens that stimulate the activation and proliferation of endothelial cells. Altogether, these data increase knowledge about a murine model that is of interest for investigating the pathology of the schistosomiasis and also the dynamics of hematopoiesis.

## Introduction

Schistosomiasis is a parasitic disease commonly related to environment factors and underdevelopment and mainly affects tropical and subtropical areas of the planet (1). It is caused by the helminth of the Trematoda class and genus Schistosoma. The only species that occurs in Brazil is *Schistosoma mansoni*, capable of causing the severe form of the disease with hepatosplenic involvement (2).

The adult worms of *Schistosoma mansoni* inhabit portal and mesenteric veins (3), where they copulate and female initiates oviposition (4). To complete the cycle, eggs need to pass through the intestinal wall and are passed in the feces. However, the blood flow carries part of the eggs and some of them are mainly trapped in liver, spleen and intestines, and stimulate a granulomatous reaction (3).

The deposition of collagenous fibers both in the granuloma and around the vessels of the portal system is responsible for the fibrosis, the main pathological manifestation of schistosomiasis (5, 6). In schistosomal infection, this mechanism may be related to angiogenesis both during the fibrosis formation and after the end of infection, helping to resolve the fibrosis (7, 8).

When mature, hepatic granulomas have three zones: the central, essentially macrophagic; the medial, which is rich in extracellular matrix; and the peripheral, where the arrangement of collagen fibers forms a loose network which harbors extramedullary hematopoiesis (9). This proliferation of hematopoietic cells outside the bone marrow can be observed under physiological and other pathological conditions. In the first case, it is usually related to hematopoietic sites that occur during embryonic development of most vertebrate (10, 11). In the second one, extramedullary hematopoiesis is frequently observed in the spleen and liver of individuals with hematological diseases, such as thalassemia and sickle cell anemia, and infectious diseases, such as some disorders caused by bacteria and murine schistosomiasis mansoni (12–14). Many years ago, some studies, including one from our research group, described the occurrence of extramedullary hematopoiesis in the peripheral zone of hepatic granulomas (15). There, we showed islands of hematopoiesis in the periphery of granulomas, composed by cells of the same myeloid lineage and in different stages of development, together with some mitotic figures, using histopathology and brighfield microscopy techniques (15). Here, we revisit the topic of hepatic extramedullary hematopoiesis in murine schistosomiasis mansoni, using current histology, immunohistology and microscopy tools, and interpreting their findings in light of modern concepts about hematopoiesis and its correlation with angiogenesis.

## Materials and methods

### Infection

The study was reviewed and approved by the Institutional Ethics Committee for Animal Research of the Oswaldo Cruz Institute (CEUA IOC, license: L-2/13). Five-day-old male Swiss Webster mice were infected by percutaneous exposure to 70 cercariae of the Belo Horizonte strain of *S. mansoni*. Four infected animals were euthanized at 35, 40-, 45-, 50- and 60-days post infection (dpi), along with two control animals. The infection was confirmed by searching schistosoma eggs in the feces of the rodents by Hoffman parasitological technique. The euthanasia was performed by initial administration intraperitoneal injection of Ketamine-Xylazine (120mg/kg and 20mg/kg) and subsequently after finding the loss of the foot reflex, the application was performed injection of 2.5% sodium thiopental (300mg/kg). The experiments were carried out in triplicate.

### Paraffin sections and histological stains

Four fragments from different liver lobes with approximately 3mm thick were collected and fixed in Carson’s Formalin-Millonig (16) for 48 hours at room temperature, washed and processing according to standard histological techniques for paraffin embedding. Sections of 5 μm thick (one/block) were obtained from paraffin blocks and stained with hematoxylin-eosin, Gomori’s reticulin, Picrosirius (17), Sirius red pH 10,2 (18), and Alcian Blue pH1,0. The slides were analyzed by brightfield microscopy using a Axioscope microscope (Zeiss, Germany) equipped with a Axiocam HRc camera (Zeiss, Germany).

### Frozen sections

Another four fragments from different liver lobes with approximately 3mm thick were collected and cryopreserved in OCT compound (Tissue Tek) and kept at -20°C. Sections of 5 μm thick were obtained in a cryostat at -20°C and stored at the same temperature.

### Immunofluorescence assay

At least two hours after being obtained, slides from paraffin blocks were deparaffinized, hydrated, washed, and hydrated in PBS. Antigen retrieval was performed in citrate buffer pH 6.0, in Pascal Chamber (Dako, USA) according to the manufacturer’s recommendations. In some cases, slides were incubated with blocking solution (2% skim powdered milk, 2.5% bovine serum albumin (Sigma, cat. A2153-100G) and 8% fetal bovine serum (Hyclone, cat. SH30088.03)) in a humid chamber for 30 min at room temperature, washed with PBS and then kept overnight with the following primary antibodies: Von Willebrand Factor (vWF, Cell Marque, cat. 250A -15), CD31 (Abcam, cat. ab28364), Fall 3 (Pharmingen, cat. 01581), VEGF (ThermoFisher, cat. RB-9031-P1), Lyve1 (Abcam, cat. ab17917), Sca-1 (Abcam, cat. ab25031), MMP9 (Abcam, cat. ab38898), Fibronectin (Abcam, cat. ab2413), and Ki67 (Abcam, cat. ab15580).

The slides were further incubated with the 1:750 secondary antibody Alexa Fluor 488 goat anti-rabbit IgG, or Alexa Fluor 594 goat anti-mouse IgG for 1h at 37°C, washed, stained with 1:10,000 Evans Blue, counterstained with 1:5,000 DAPI (ThermoFisher, cat. 03571) and mounted with ProlongGold (ThermoFisher, cat. P36934).

Slides from frozen sections were fixed in acetone at -20°C for 15 min and allowed to dry at room temperature for 15 min. They were washed in PBS and incubated with the blocking solution previously described for 40 min, and then proceeded for the antibodies incubation as described.

The slides prepared for immunofluorescence were analyzed on a LSM 710 confocal microscope (Zeiss, Germany).

## Results

### Morphological evolution of liver lesions

**Figure 1:**
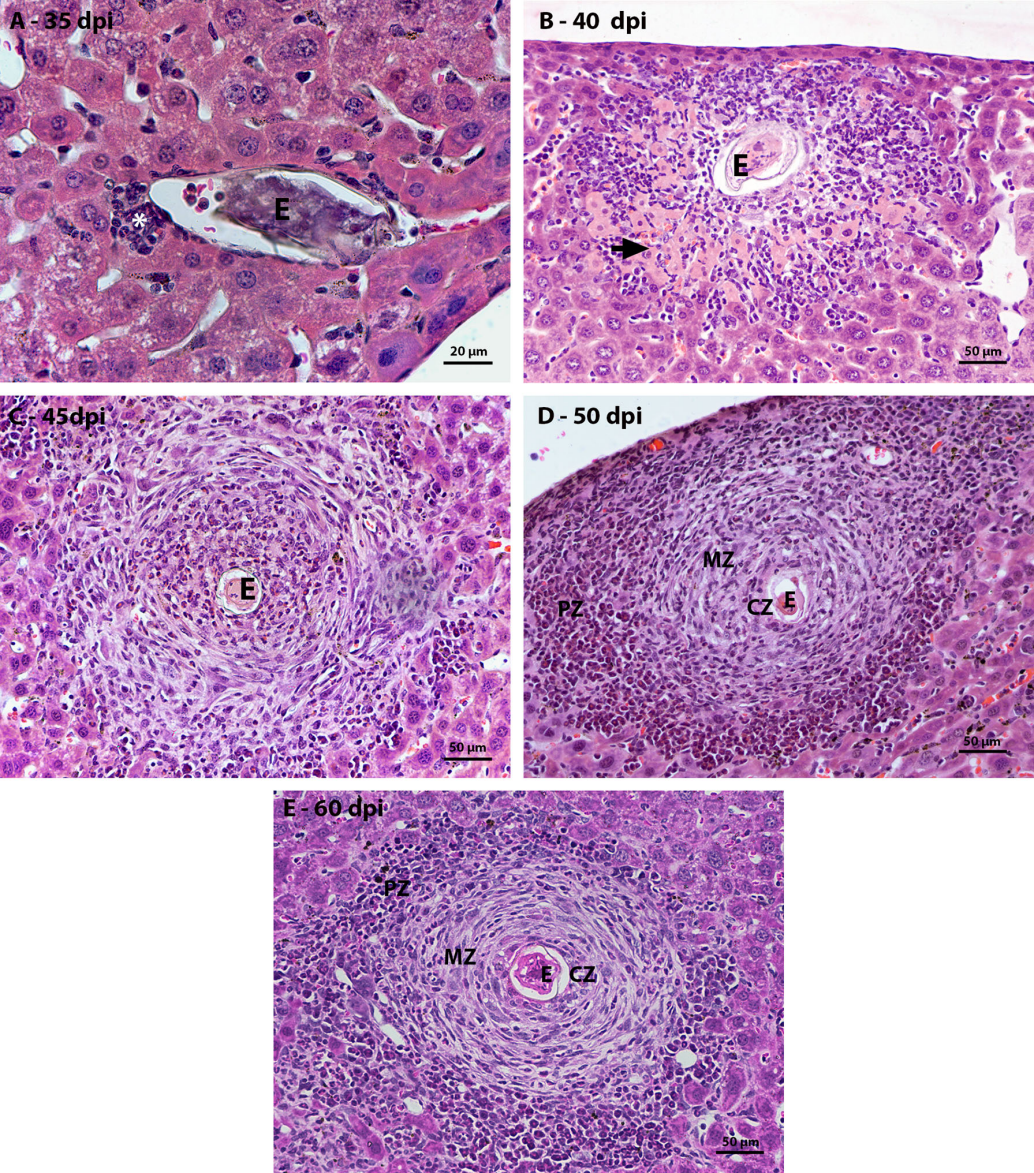
Figure 1 Evolution of hepatic granulomatous lesions over the days of infection. HE-stained sections demonstrate: **(A)** a small inflammatory infiltrate (*) composed of eosinophils and macrophages around the egg **(E)** at 35 dpi; **(B)** inflammatory infiltrate (*) greater than that observed at the previous point of infection, located between the necrotic hepatocytes (arrow) around the egg (O); **(C)** Larger granuloma with deposition of fibrous extracellular material, but still disorganized at 45 dpi; (**D**, **E**) Granulomatous lesion found in the acute phase of infection, at 50 and 60 dpi, is organized into three zones – central (CZ), medial (MZ) and peripheral (PZ), with proliferation of hematopoietic cells in the peripheral zone. Bars, A: 20 µm, B – E: 50 µm.

The oviposition of the adult worm starts around the thirtieth day of infection and continues during all the life of the adult worm. At 35 dpi, it was possible to identify an average of three units of parasite eggs per histological section in the liver. These eggs had a small inflammatory infiltrate located between the hepatocytes and the blood vessel wall and were composed mainly of macrophages and eosinophils (**Figure 1A**). With the development of the infection, at 40 dpi, there was an increase in the number of cells that made up the inflammatory infiltrate around the egg. These inflammatory cells, in some cases, were located between necrotic hepatocytes (**Figure 1B**). At 45 days of infection, it was possible to observe some monocytes and eosinophils in the region closest to the egg. Around this area there were some elongated cells among extracellular matrix. At this point, although the granulomas were still immature (**Figure 1C**).

The first mature granulomas, with central, medial, and peripheral zones, were found at 50 and 60 dpi. At this time, the granulomas that had this conformation showed proliferation of hematopoietic cells in the peripheral zone. The zone of extramedullary hematopoiesis was composed of cells of the myeloid lineage, mainly eosinophils, neutrophils and monocytes, in different stages of maturation (**Figures 1D, E**).

**Figure 2:**
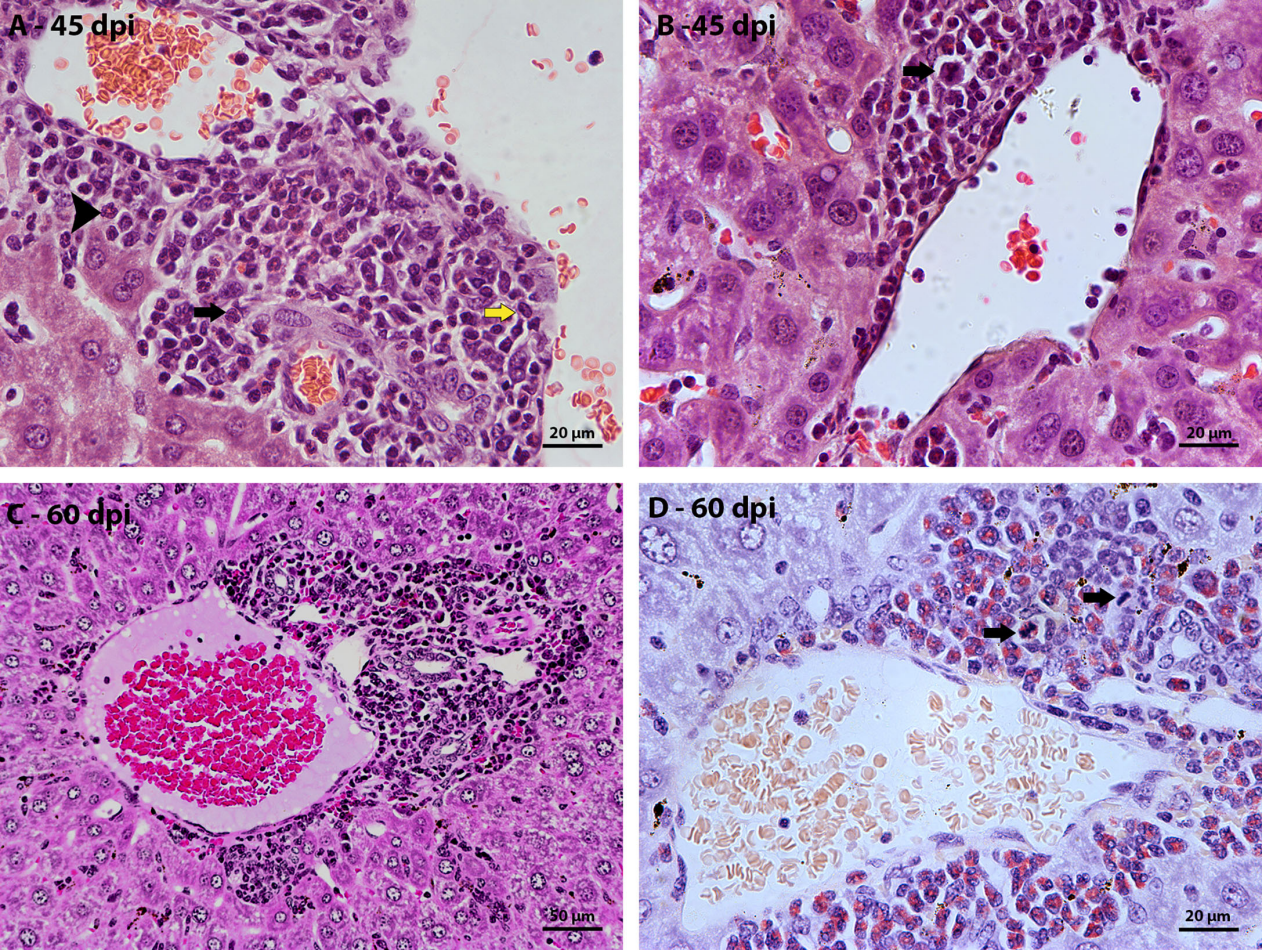
Morphological aspect of hepatic perivascular lesions in mice infected with *S. mansoni*. HE-stained sections demonstrate: **(A)** Inflammatory infiltrate composed of myeloid cells, eosinophils (arrowhead), macrophage cells (yellow arrow) and neutrophils (black arrow) in the hepatic portal space of mice at 40 dpi. **(B)** hematopoietic cells of the myeloid lineage, highlighting a cell in mitosis (arrow), in the periphery of a vein at 45 dpi; **(C)** Hepatic portal space with many mature and immature hematopoietic cells at 60 dpi; and **(D)** highlighting cells in mitosis (arrow) in the periphery of vessels in the hepatic portal space at 60 dpi. Bars, **(A, B, D)** 20 µm, **(C)** 50 µm.

The largest hepatic vessels, such as central vein, portal vein and portal arteriole, without a parasitic element nearby, also had inflammatory infiltrates around them. From 35 dpi, cells of the myeloid lineage located between the vessel wall and the hepatocytes were observed. At 40 dpi there was an increase in the amount of surrounding inflammatory cells, with the presence of eosinophils, neutrophils, and monocytes (**Figure 2A**). Cells in mitosis were observed in the periphery of these vessels at 45 days of infection (**Figure 2B**). The structural organization of the distribution of hematopoietic cells around the vessels is similar to that of the previous points of infection. The main difference is the increase in more immature myeloid cells and in mitosis.

The morphological description of the liver lesions during schistosomal infection pointed to the evolution of the granulomatous structure over the days of infection with definition in three zones, the identification of the peripheral zone of mature granulomas and the periphery of the large vessels as favorable regions for the establishment and maintenance of extramedullary hematopoiesis. The proliferation of hematopoietic cells in these regions has been described from the frequently observed mitoses. Considering the ephemerality of cell division in these cases, it was important to associate other techniques for identifying proliferating cells to improve the topographical and kinetic description of hepatic extramedullary hematopoiesis during schistosomal infection.

### Identification of proliferating cells

**Figure 3:**
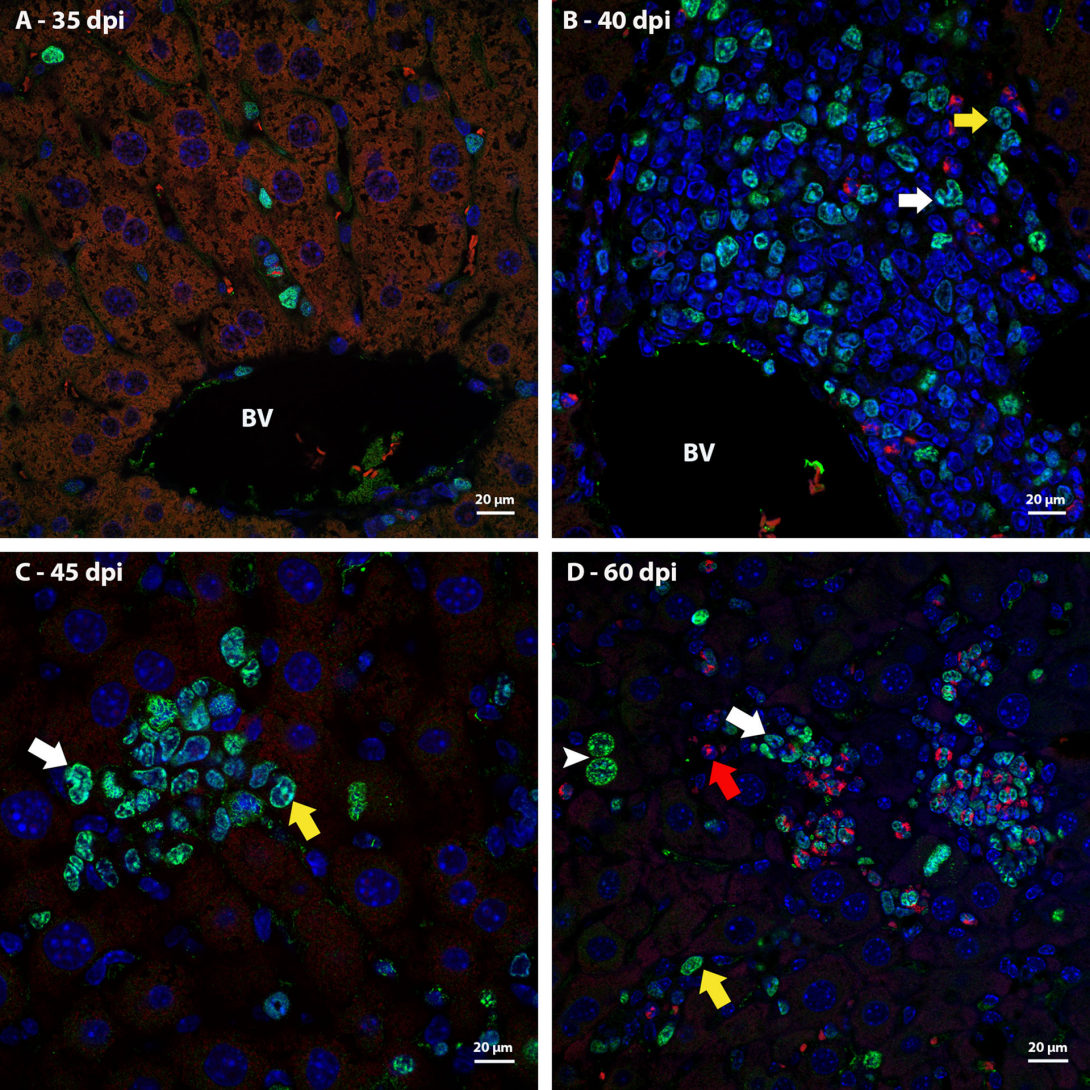
Localization of proliferating cells in the periphery of blood vessels in the liver by Ki67 labeling. **(A)** Some Ki67-positive cells are seen into the sinusoids at 35dpi; **(B)** cells of the neutrocytic (white arrow) and monocytic (yellow arrow) lineage that express Ki67 are located among other myeloid cells around a blood vessel at 40 dpi; **(C)** Ki67-positive neutrophils (white arrow) and monocytes (yellow arrow) within a sinusoid at 45 dpi; **(D)** cells of the eosinophilic (red arrow), neutrocytic (white arrow) and monocytic lineages (yellow arrow) in different stages of differentiation that expressing Ki67 are located inside of sinusoid at 60 dpi. At this point of infection also is possible to observe some hepatocytes expressing the molecule which indicates cell proliferation (arrowhead). Bar: 20 µm. Blue: DAPI, Red: Evan’s blue, Green: Ki67.

**Figure 4:**
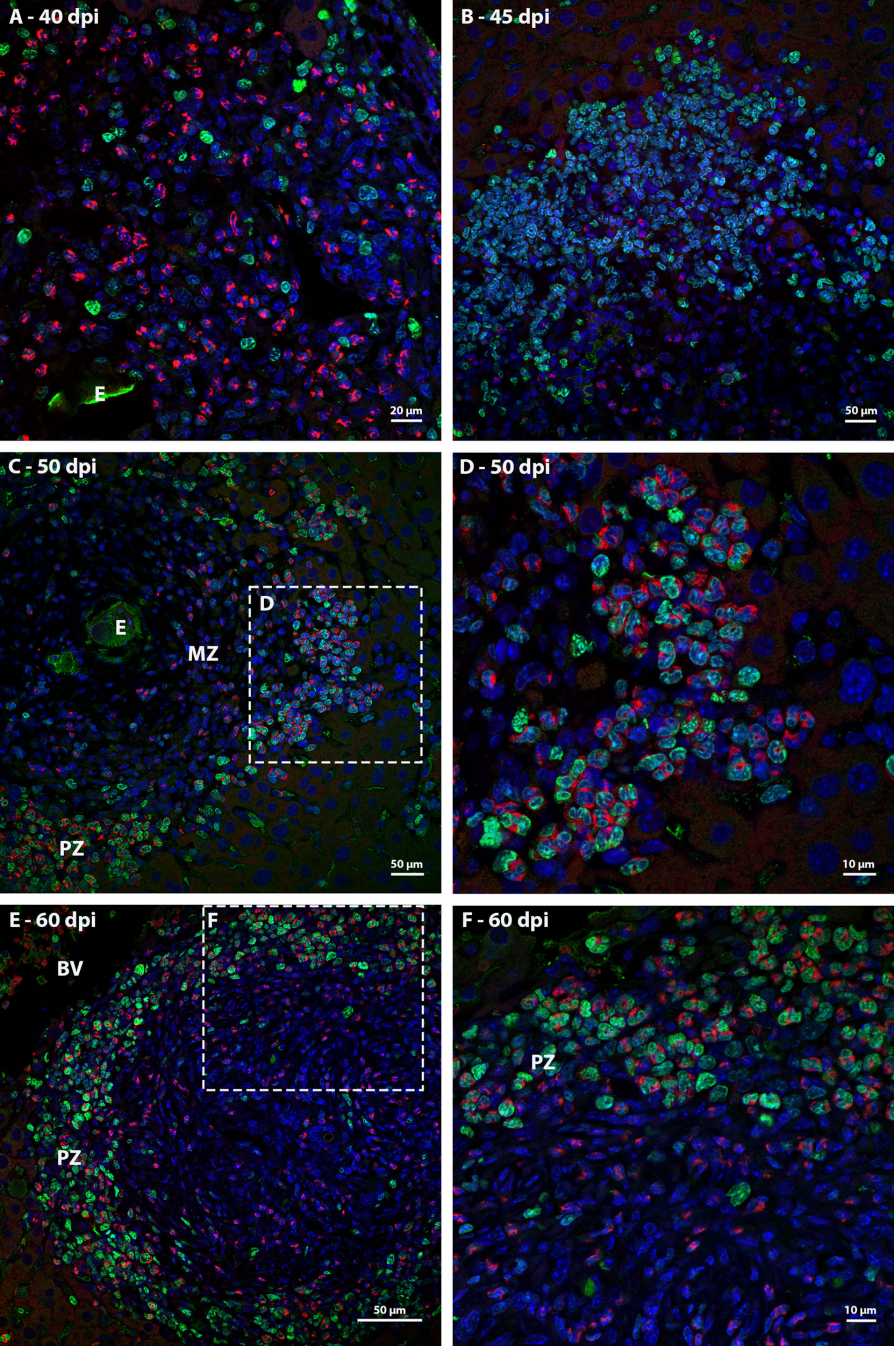
Localization of proliferating cells in schistosomal granuloma in the liver by Ki67 labeling. **(A)** Cells of the neutrocytic and monocytic lineage which express Ki67 are arranged among the other inflammatory cells that form the 40-day infection granuloma **(B)** A greater number of hematopoietic cells, mainly of the neutrocytic and monocytic lineage, are stained with Ki67 and are concentrated in the periphery of the granuloma at 45 dpi; **(C, D)** At 50 dpi in addition to cells of the monocytic and neutrocytic lineages, cells of the eosinophilic lineage expressing Ki67 are also observed. These cells are in the peripheral zone of the mature granuloma; **(E, F)** The proliferate Ki67-labeling hematopoietic cells are localized in the peripheral zone of granuloma at 60 dpi, as was observed in the previous point of infection analyzed. Bars, **(A)** 20 µm, **(B, C, E)** 50 µm, **(D, F)** 10 µm. Blue: DAPI, Red: Evan’s blue, Green: Ki67.

Ki67 immunostaining was used to confirm the data on cell proliferation, obtained by the analysis of mitotic cells, mainly hematopoietic. At 35 dpi, Ki67 positive myeloid cells were found within sinusoids in a region near to a hepatic vessel (**Figure 3A**). At 40 and 45 dpi, there was an increase in the number of hematopoietic cells expressing Ki67, especially at 45 dpi. These cells were present in the periphery of large vessels, inside granulomas and within sinusoids and belonged mainly to neutrophilic and monocytic lineages (**Figures 3B, C**; **Figures 4A, B**). At 50 and 60 dpi, there were cells of monocytic, neutrophilic and eosinophilic lineages positive for Ki67 around large vessels, as well as within sinusoids and granulomas. In the latter case, the location of the cells was restricted to the peripheral zone (**Figure 3D**; **Figures 4C–F**).

**Figure 5:**
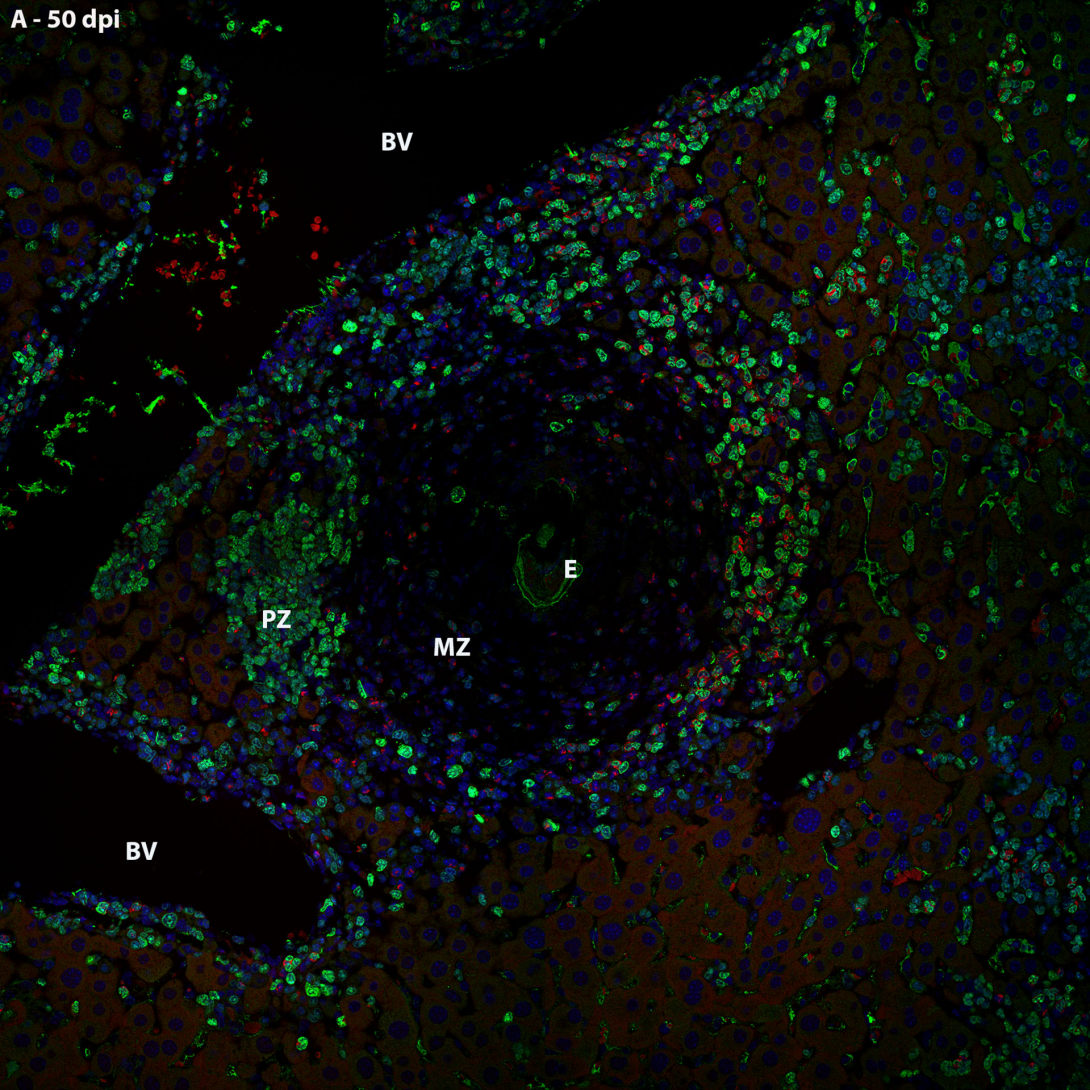
General localization of proliferating cells of murine schistosomal liver by Ki67 labeling, during acute phase of the disease. A Hematopoietic cells proliferating in the schistosomal liver of mice are mainly and almost restrictedly located in the periphery of the large vessels, inside some sinusoids and in the peripheral zone of mature granulomas. E, Egg; BV, Blood Vessel; MZ, Medial Zone; PZ, Peripheral Zone. Blue, DAPI; Red, Evan’s blue; Green, Ki67.

Thus, it was possible to observe that the location of proliferating hematopoietic cells in the murine schistosomal liver varies according to liver lesions. During the acute phase the proliferating cells are almost restricted to the peripheral zone of the granulomas, around the large vessels and within some sinusoids (**Figure 5**).

### Extracellular matrix elements associated with extramedullary hematopoiesis

**Figure 6:**
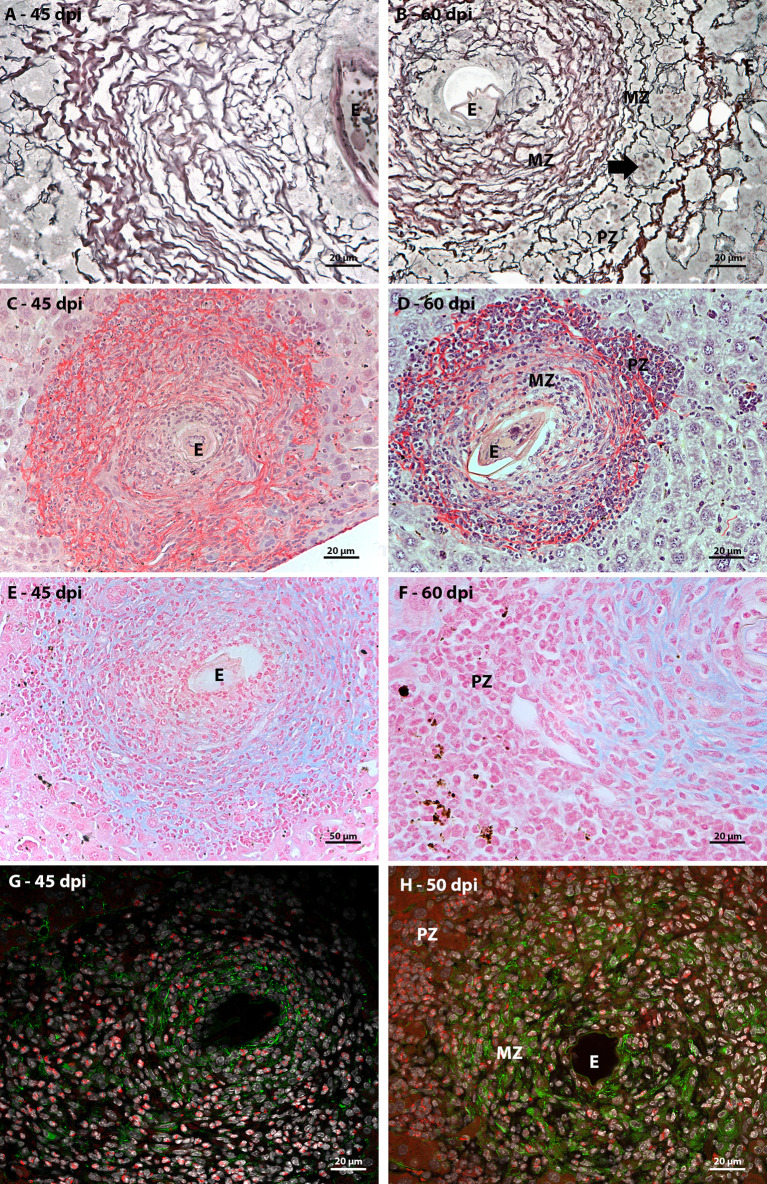
Extracellular matrix elements found in hepatic schistosomal granuloma. **(A)** Reticular fibers, highlighted in black by Gomori’s reticulin staining, extend throughout the immature granuloma; **(B)** The reticular fibers, evidenced by Gomori’s reticulin staining, in the 60 dpi granuloma form a looser mesh-like in the peripheral zone (PZ) of the granuloma, forming compartments (arrow) that house hematopoietic; **(C)** Types I and III collagen fibers are evidenced by Sirius red staining and are present among most of the cells that are part of the granuloma of 45 dpi, with the exception of the closest to the egg **(E)**; **(D)**The collagenous fibers identified by Sirius red staining present in the mature granuloma have the conformation of a network with the looser weft in the peripheral zone (PZ) of the structure. In this region, these thin fibers form small compartments that house isolated hematopoietic cells or in small groups; **(E)** Sulfated acid glycoconjugates can be identified by Alcian Blue staining in the full extent of immature granulomas; **(F)** Alcian blue staining is concentrated in medial zone and is absent in the peripheral zone of mature granulomas; **(G)** In immature granuloma, fibronectin expression occurs more concentrated in the region closest to the egg and more diffused among cells that are distant from the parasitic element; **(H)** Fibronectin expression in mature granuloma can be seen in the medial zone (MD) and is absent in the peripheral zone (PZ). Bars **(C–E)**: 50 µm, **(A, B, F, G, H)** 20 µm. Stains: **(A, B)** Alcian Blue pH 1,0, **(C, D)** Picrosirius, **(E, F)** Gomori’s Reticulin. **(G, H)** White: DAPI, Red: Evan’s blue, Green: Fibronectin.

**Figure 7:**
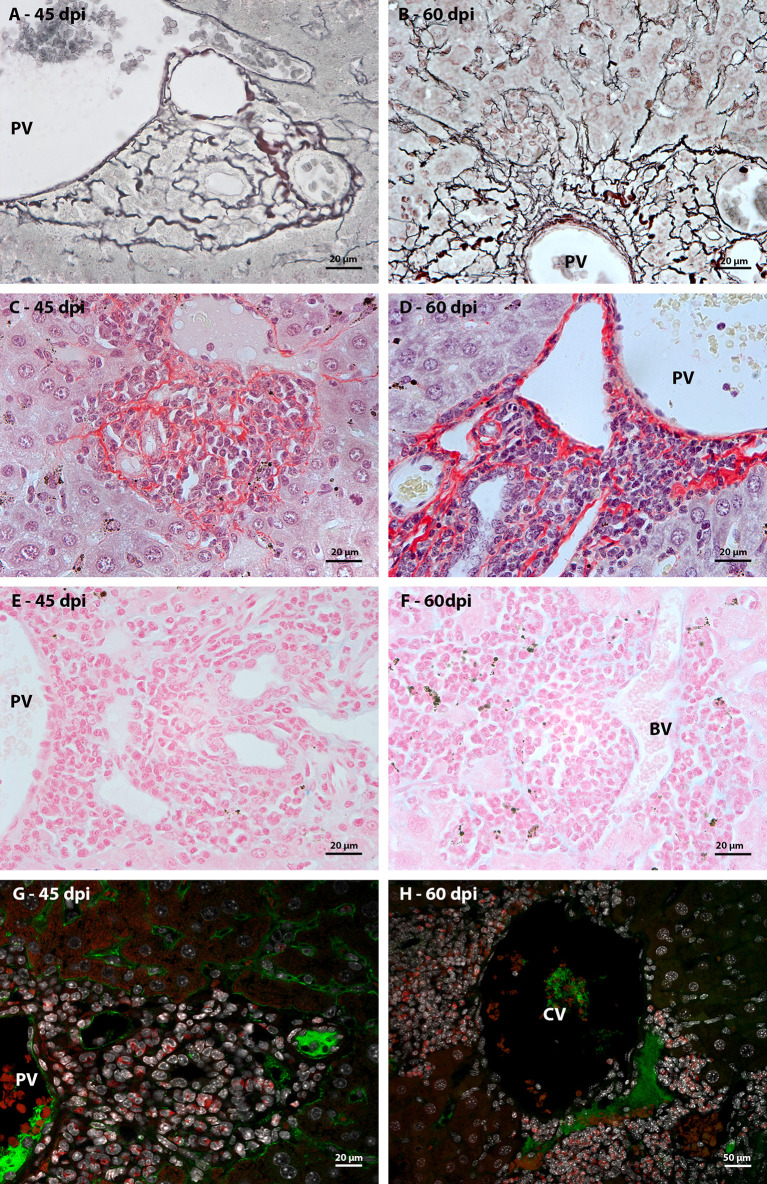
Extracellular matrix elements found at the periphery of large hepatic vessels during *Schistosoma mansoni* infection. **(A, B)** the arrangement of reticular fibers, highlighted by Gomori’s reticulin, was resembling to the observed in samples stained by picrosirius in both of point of infection; **(C)**, **(D)** At different times of infection, types I and III collagen fibers, identified by picrosirius, are arranged in a similar morphology among the hematopoietic cells that surround the large hepatic vessels. They form compartments like those seen in the periphery of mature granulomas, which harbor groups of hematopoietic cells; **(E)** Absence of Alcian Blue staining, which identifies sulfated acid glycoconjugates, among hematopoietic cells present in the periphery of large hepatic blood vessels (BV), such as portal veins (PV), at earlier points of infection; **(F)** and during the acute phase of the disease; **(G, H)** Anti-fibronectin immunostaining did not identify the expression of the molecule among the hematopoietic cells that surrounded the hepatic blood vessels at the two points of infection (45 and 60 dpi). Bars, **(A–G)** 20 µm, **(H)** 50 µm. Stains: **(A, B)** Alcian Blue pH 1,0, **(C, D)** Picrosirius, **(E, F)** Gomori’s Reticulin. **(G, H)** White: DAPI, Red: Evan’s blue, Green: Fibronectin.

In order to characterize the microenvironment favorable to this event, it was important to investigate the stroma of these regions, with special attention to some extracellular matrix elements. Considering the fibrosis present in the disease and the importance of collagen fibers for the organizational structure of the granuloma, picrosirius staining and Gomori’s Reticulin histochemistry were performed. In both analyses, it was possible to observe the uniform distribution of fibers throughout the immature granuloma (**Figures 6A, C**) and the contribution of these structures to the organization of the mature granuloma into three zones. In the peripheral zone of mature granulomas, these reticular or collagen fibers were arranged in a loose-mesh-like that surrounded groups of hematopoietic cells forming small compartments (**Figures 6B, D**). The analysis of the large vessels that had surrounding hematopoietic cells also showed the presence of these compartments formed by collagen fibers. However, in this case, there was no difference in the perivascular structural organization between 45 and 60 dpi (**Figures 7A–D**).

Alcian Blue staining pointed to the presence of acidic and sulfated glycoconjugates. They were identified in the full-length immature granulomas (**Figure 6E**). In the periphery of the large vessels, both in the earliest points (**Figure 7E**) and in the acute phase of the disease (**Figure 7F**), staining was absent among the hematopoietic cells. The absence of staining was also identified in the peripheral zone of mature granulomas (**Figure 6F**). Fibronectin expression showed a morphological pattern similar to the presence of sulfated acid glycoconjugates. The molecule was immuno-identified in the entire extension of the early granulomas (**Figure 6G**) and absent among the hematopoietic cells that surrounded the great vessels throughout all times of infection (**Figures 7G, H**) and in the peripheral zone of the mature granulomas (**Figure 6H**).

The microenvironment favorable to the maintenance of hematopoietic cell proliferation seems then to be dependent on the formation of compartments delimited by collagenous fibers and independent of sulfated acid glycoconjugates and fibronectin.

### Extracellular matrix remodeling and possible chemoattraction of hematopoietic cells

**Figure 8:**
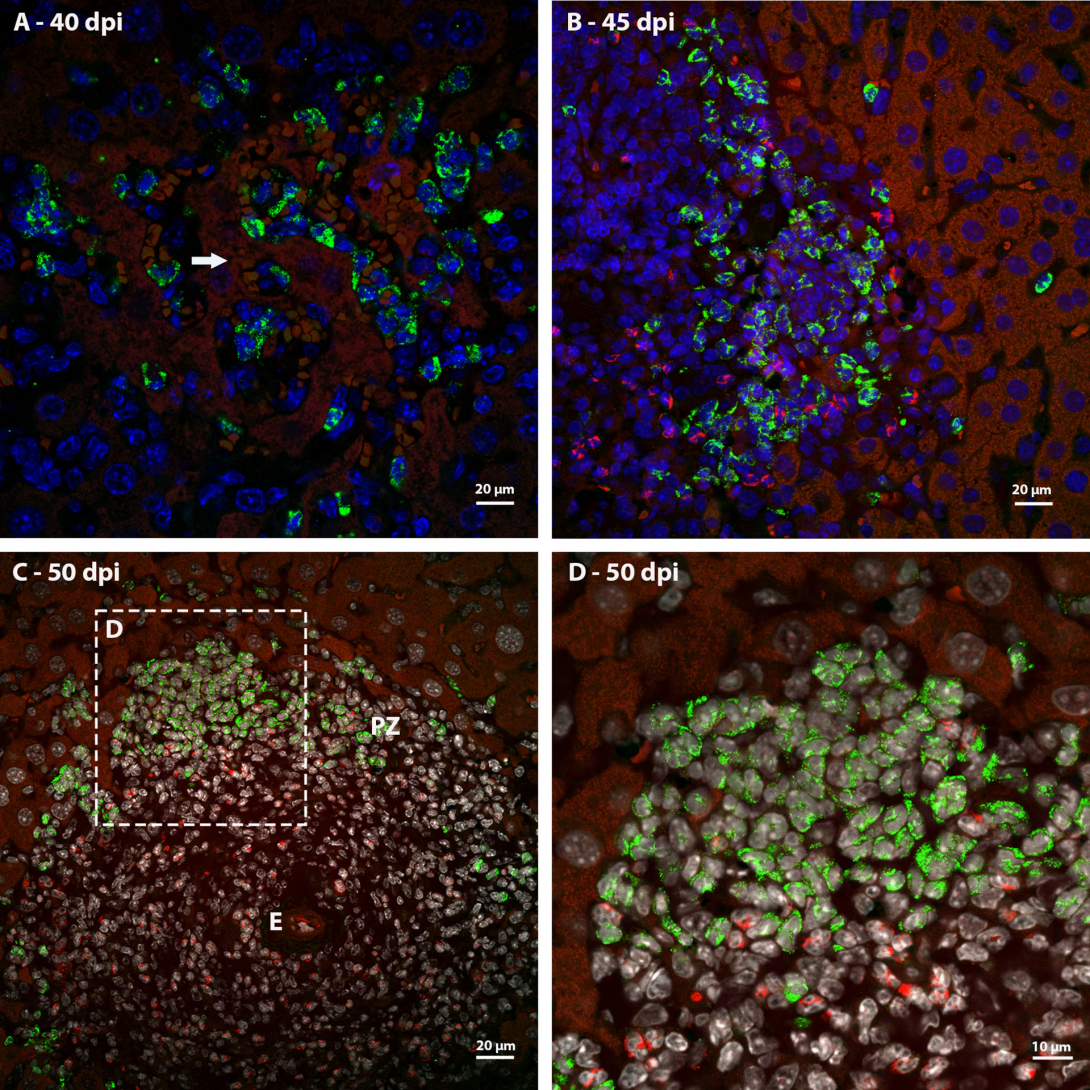
Identification of expression of MMP9 in murine liver granulomas by immunofluorescence. **(A)** Neutrophils expressing MMP9 were immunolabeling in an inflammatory infiltrate between necrotic hepatocytes (arrow) at 40 dpi; **(B)** The expression of MMP9 in granulomas found in mice at 45 dpi was concentrated in neutrophils located in de periphery of structure; **(C, D)** Neutrophils at different points of infection express MMP9 preferentially in the peripheral zone of mature granulomas at 50 dpi. Bars, **(A–C)** 20 µm, **(D)** 10 µm. DAPI: blue **(A, B)**, white **(C, D)**, Evan’s blue: red, MMP9: green.

**Figure 9:**
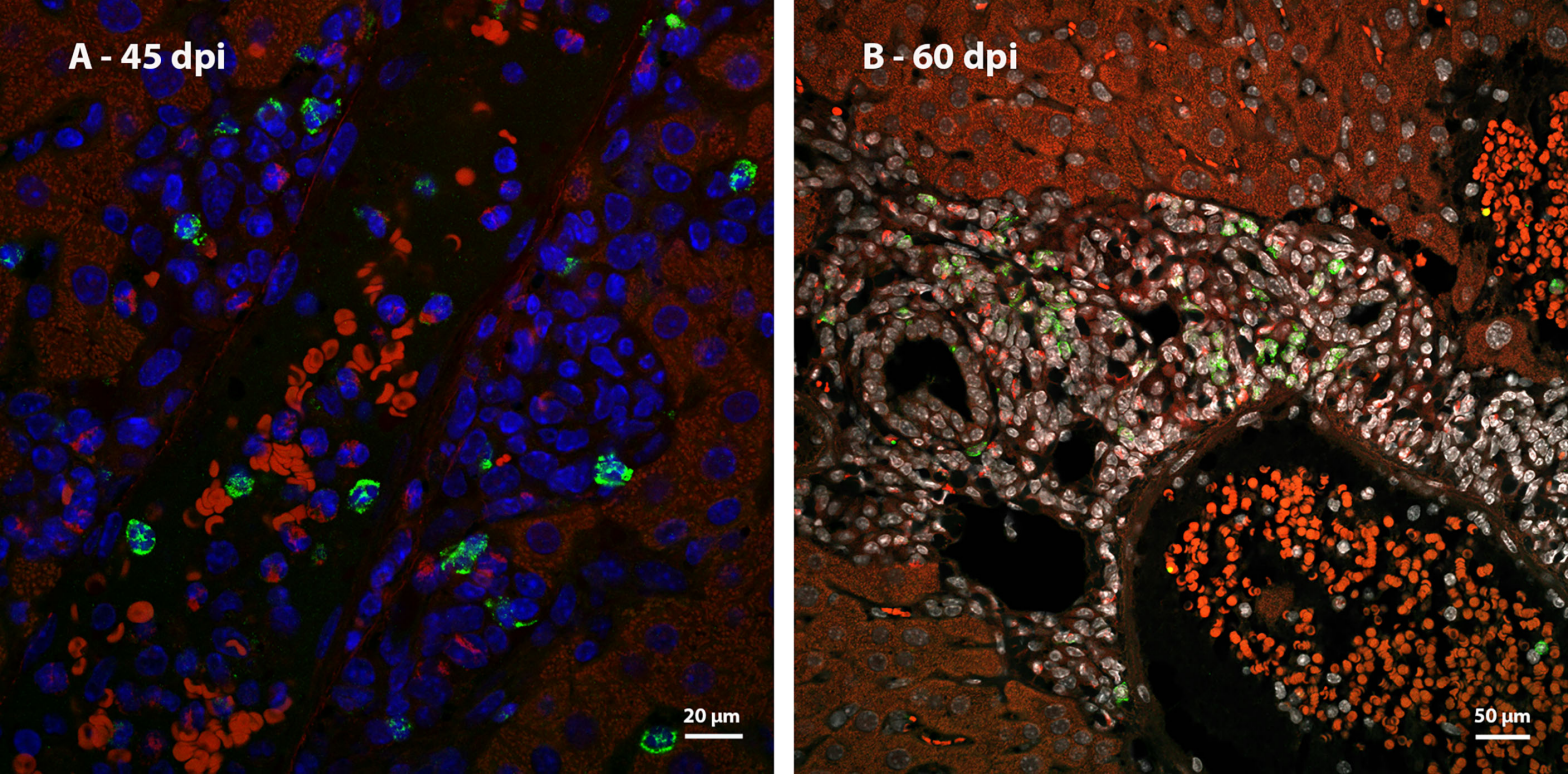
Identification of expression of MMP9 in the periphery of large blood vessels in mice infected by *S. mansoni* by immunofluorescence. **(A)** Some neutrophils expressing MMP9 are arranged randomly among others hematopoietic cells in the periphery of a vessel with no apparent parasitic element at 45 dpi and **(B)** 60 dpi. Bars, **(A)** 20 µm, **(B)** 50 µm. DAPI: blue **(A)**, white **(B)**, Evan’s blue: red, MMP9: green.

Immunostaining identified metalloproteinase 9 (MMP9) in neutrophils present among necrotic hepatocytes at the first points of infection (**Figure 8A**). In granulomas, cells immunoreactive for this molecule were preferentially located in the peripheral zone, forming small groups at the interface with hepatocytes (**Figures 8B–D**). In the periphery of the vessels with surrounding hematopoietic cells, the neutrophils positive for the molecule did not form clusters but were arranged in a dispersed manner (**Figures 9A, B**).

The organization of the granuloma structure seems to favor the presence of clusters of cells that express MMP9 and this does not occur in the periphery of the great vessels.

### Identification of immature hematopoietic cells in the liver

**Figure 10:**
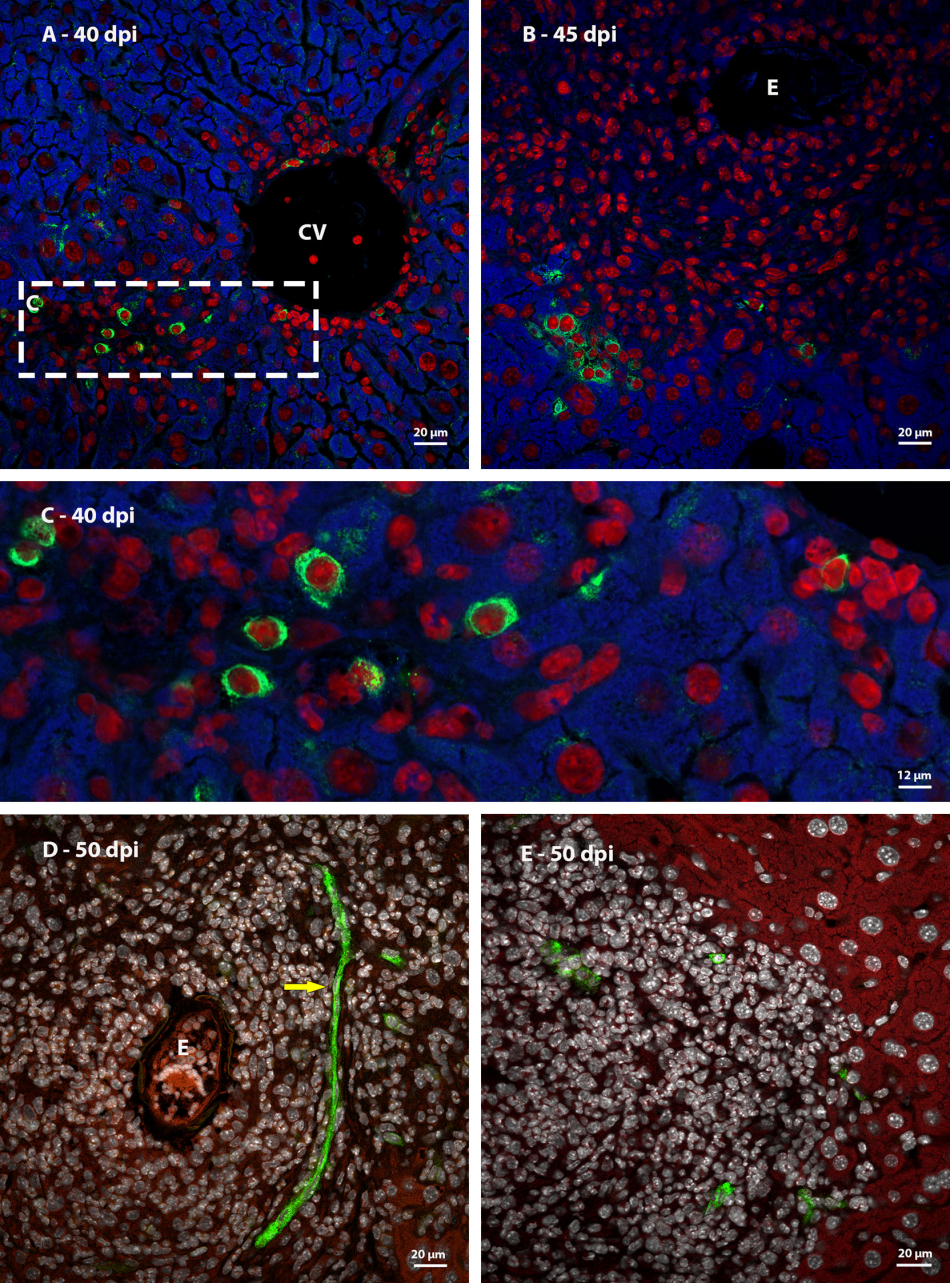
Identification of immature hematopoietic cells by immunofluorescence in livers of mice infected by *S. mansoni.* **(A, C)** Fall 3 immunostaining identified cells with characteristics of immature hematopoietic cells (loose chromatin and protruding nucleolus) close to a central vein (CV); **(B)** These cells Fall 3-positives also were found in the periphery of 45 dpi granuloma; **(D, E)** The immunostaining identified the expression of Sca-1 in a blood vessel (arrow) placed on medial zone some cells located in the peripheral zone of 50 dpi granuloma. Bars **(A, B, D, E)** 20 µm, C: 12 µm DAPI: red **(A–C)**, white **(D, E)**, Evan’s blue: blue **(A–C)** and red **(D, E)**, Fall 3: green **(A–C)**, Sca-1: green **(D, E)**.

Considering the possibility of mobilization of immature hematopoietic cells from the bone marrow to the liver, the degree of maturation in which the hematopoietic cells from the periphery of the granulomas and the great vessels presented was investigated. During the labeling of Fall-3, the analysis identified groups of hematopoietic cells close to large vessels and in the periphery of the granulomas with characteristics of immature hematopoietic cells. These cells had loose chromatin, protruding nucleolus, and larger size (**Figures 10A–C**).

Some isolated cells were found in the periphery of mature granulomas expressing Sca-1, indicating the presence of immature cells in this region. In addition, some blood vessels, including intragranulomatous ones, also expressed the molecule.

Phenotypic characteristics provided by morphological analyzes and the identification of the expression of a molecule present only in more immature hematopoietic cells suggest the migration of immature hematopoietic cells, which establish and proliferate in the schistosomal liver.

### Phenotypic analysis of hepatic blood vessels

**Figure 11:**
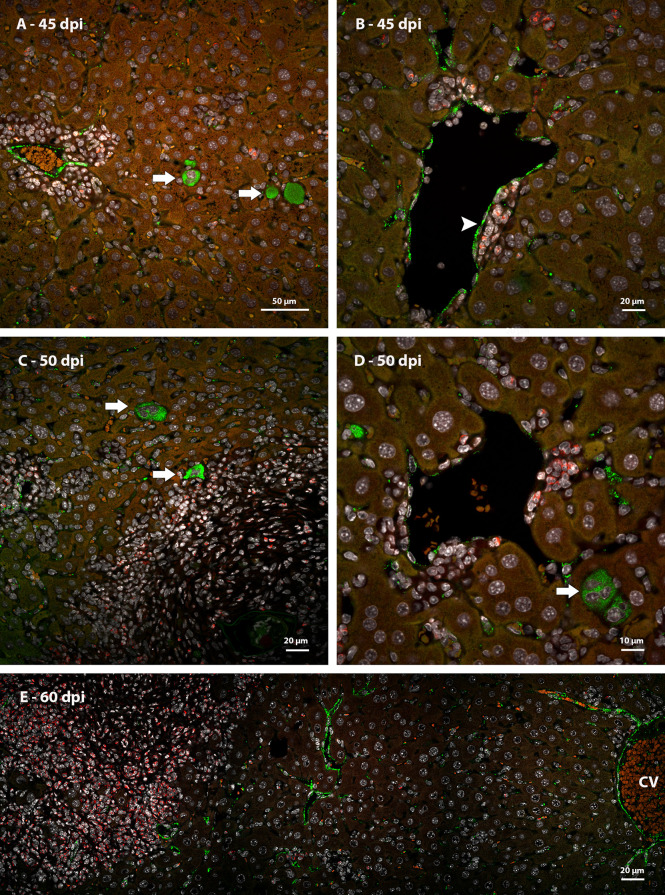
Expression of vWF in livers of mice infected by *S. mansoni*, identified by immunofluorescence. **(A)** Detection of more than one megakaryocyte among the hepatocytes in the same area of analysis at 45 dpi; **(B)** Blood vessel vWF-positive with inflammatory cells in the periphery situated between the endothelium and the hepatocytes at 45 dpi; **(C) (C)** Megakaryocytes (arrow) are still found in the liver of infected animals during the acute phase of the disease, close to the peripheral zone of the granulomas, but still in contact with hepatocytes, **(D)** and close to a vessel with surrounding hematopoietic cells at 50 dpi; **(E)** central vein (CV), vessels associated with the periphery of the granuloma and some sinusoids express the protein at 60 dpi. Bars, **A**: 50 µm, **(B, C, E)** 20 µm, D: 10 µm. DAPI: white, Evan’s blue: red, vWF: green.

Immunoreactivity to vWF was present in some rare sinusoids, in portal and central veins when they were associated with the parasite egg, or with surrounding inflammatory infiltrates (**Figures 11A, B, D, E**) and, in some cases, in wide-lumen blood vessels present in the peripheral zone of mature granulomas (**Figure 11E**). In addition, vWF is also capable of identifying megakaryocytes. These cells were found between hepatocytes, close to the periphery of the granuloma (**Figures 11A, C**) or around large vessels with surrounding hematopoietic cells (**Figure 11D**), at all points of infection analyzed. In some cases, these cells were present in more than one unit per field.

**Figure 12:**
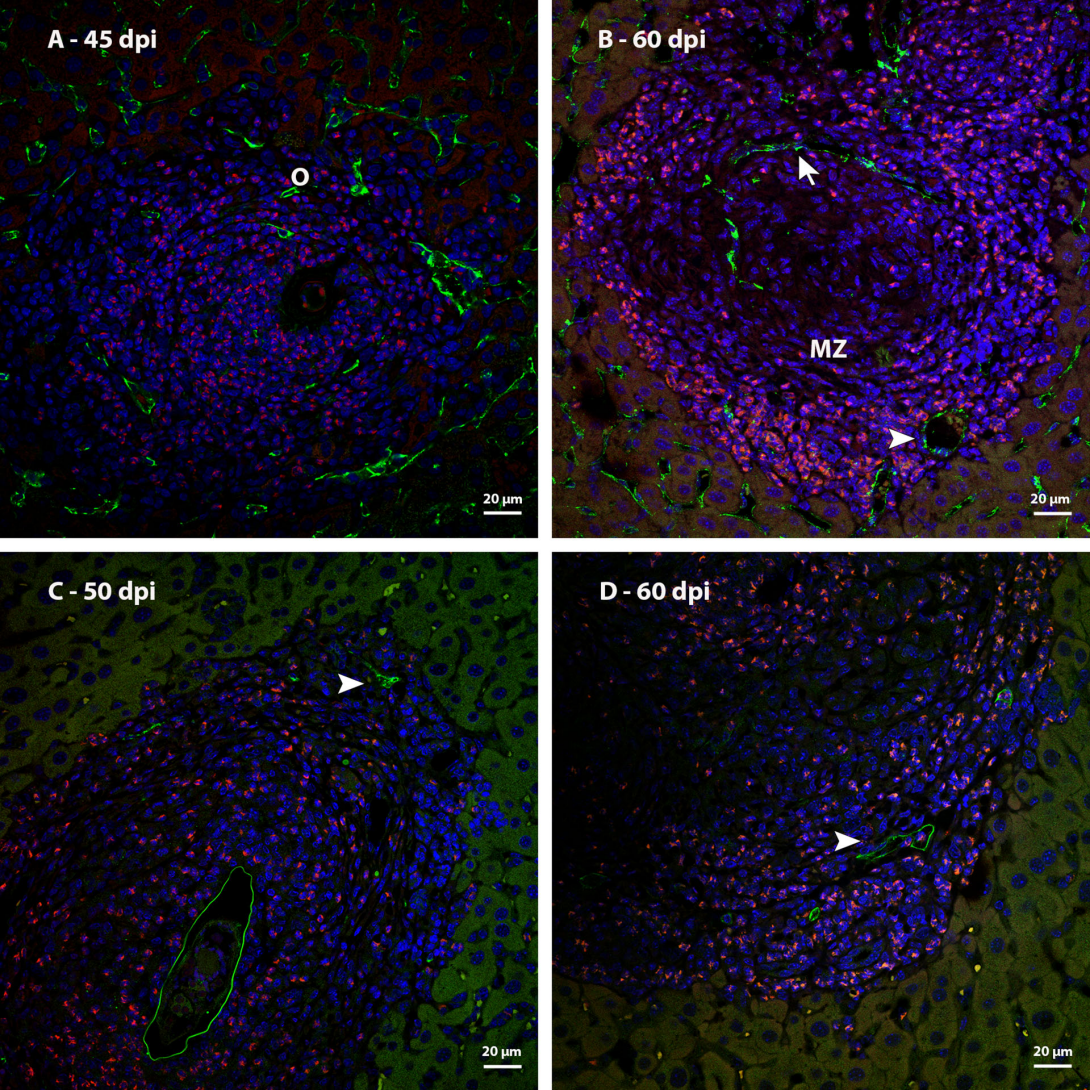
Identification of blood vessels in livers of mice infected by S. mansoni, immunostaining by CD31 and Lyve 1. **(A)** Sinusoids that are observed among the other cells that form the granuloma at 45dpi express CD31; **(B)** CD31 immunostaining identified a blood vessel with sinusoidal morphology (arrow) in the medial zone of the granuloma and other vessels with broad lumen (arrowhead) in the peripheral zone of the same granulomas at 60 dpi; **(C)** Identification of lymphatic vessels (arrowhead) located in the periphery of mature granulomas at 50 and **(D)** 60 dpi. Bars, 20 µm. DAPI: blue, Evan’s blue: red, CD31: green **(A, B)**, Lyve-1: green **(C, D)**.

The analysis of immunostaining by CD31 in mature hepatic granulomas showed blood vessels present between the middle and peripheral zones, and others, very characteristic for having a wide lumen, in the peripheral zone (**Figures 12B**). These vessels were not identified in samples from points of infection with earlier granulomas (**Figure 12A**). The expression of Lyve-1 in some vessels present in the peripheral zone of mature granulomas suggests the appearance of vessels in this region (**Figures 12C, D**).

In the schistosomal liver, there are vessels with activated endothelium that express vWF, in addition to the appearance of new vessels in the middle and peripheral zones of mature granulomas. Some of them are immunoreactive to lymphatic vessel markers.

## Discussion

This work confirmed the occurrence of extramedullary hematopoiesis during the schistosomal infection and demonstrated the localization of the proliferating myeloid cells in the liver. We point to some phenotypic characteristics of the hematopoietic cells located in proliferating areas and produced other data, such as the distribution of extracellular matrix compounds, which can help to understand the process of attraction, establishment and maintenance of hepatic extramedullary hematopoiesis. Also, it was identified increase in the quantity of blood vessels, which can be stimulated both directly by the parasite and by mechanisms that have intersection with extramedullary hematopoiesis.

It was identified extramedullary hematopoiesis in the liver of mice infected by *S. mansoni*, as previously observed by Lenzi et al. (15). Using light microscopy, these authors detected myeloid cells in mitosis, mainly in the peripheral zone of granuloma, beginning at the 40^th^ day of infection. Here, we identified myeloid cells in mitosis both in the granuloma and around of large hepatic blood vessels and, to improve the location of the proliferating cells we used immunolabeling for Ki67, which showed proliferating myeloid cells inside the sinusoids since 35 dpi. It can indicate that some inflammatory cells which are attracted to schistosomal liver arrive in this organ in proliferation shortly after the start of oviposition, that happens around the 30^th^ day post infection (2). On the other hand, it is possible to suggest that presence of parasite, or the release of soluble eggs antigens (SEA), induces the activation of hepatic resident inflammatory cells, such as Kupffer cells. Although it was previously considered that Kupffer cells are derived from bone marrow HSC, they are actually originated from macrophage progenitors that come from yolk sac and persist in some organs during the adult phase, such as liver. These macrophage progenitors can be responsible for the local proliferation of the Kupffer cells in some cases, like inflammatory events (19, 20).

The proliferating cells which were labelled by Ki67 in granulomas have the distribution pattern related to evolution of each granuloma, that starts in disorganized immature form, the pre-granulomatous exudative stage, and evolute to a mature granuloma, which is in the exudative-productive stage (21). The last is characterized by three well defined zones: central, middle, and peripheral (9). During the exudative-productive stage, the Ki67-positive cells were almost restricted to the peripheral zone of granulomas, while on the immature ones these cells were distributed in a disorganized way around the parasite eggs, as observed by Lenzi et al. (15).

In addition to cellular proliferation in the peripheral zone of granulomas, it was also identified proliferating cells around blood vessels of portal spaces, central veins, and some sinusoids. The myeloid proliferation in these regions resembles the observed in the fetal liver of mice during the embryonic development. Between the 15^th^ and 17^th^ days post coitus occurs a peak in the eosinophilic and neutrophilic lineages production located under the hepatic capsule and around the portal veins, places where there is mesenchymal stroma (22). In opposite of what is found in mature granulomas, the distribution of proliferating cells around the hepatic blood vessels has the same aspect since the start of the oviposition. This can indicate difference in the modulation of cell proliferation in these two areas.

The extramedullary hematopoiesis described here is different from the observed in hematological disorders and in massive hepatic necrosis, hepatocarcinoma and in adenoma. In these last diseases there is erythroid proliferation among hepatocytes, which provide a endoderm-derived stroma (14, 23) while in murine schistosomiasis it was detected myeloid proliferation in microenvironments with elements of mesenchymal origin.

Hepatic schistosomal granulomas have collagen fibers and it is one of the causes of the hepatic fibrosis in the schistosomiasis (24). In mature granulomas, collagen fibers are present in a great concentration in medial zone and show a loose-mesh-like aspect in peripheral zone. Usually, these compartments were filled with cellular clusters of the same hematopoietic lineages, mainly neutrophilic, monocytic or eosinophilic ones. These data led us to consider the possibility of clonal expansion in these regions.

Although fibronectin is a structural molecule expressed by osteoblasts, pericytes and endothelial cells in bone marrow (25), with many functions in hematopoiesis such as migration, homing (26), retention (27), differentiation, and cell proliferation (28), it was not detected by immunofluorescence both in peripheral zone of the granulomas and among the hematopoietic cells around large vessels. The proliferation of myeloid cells around large vessels with presence of reticular fibers and absence of fibronectin also was observed by Ayres-Silva et al. in mice fetal liver (22). In some cases, such as hematological diseases, the adult liver can support extramedullary hematopoiesis. Some authors suggest that this mechanism happens due to hypoxia and a possible reactivation of hematopoietic stem cell (HSC) niche of the fetal liver (14, 29).

Heparan sulfate is an important glycoconjugate which can participate of regulation of retention and proliferation of HSC, through the link to SDF-1 by endothelial cells and osteoblasts (30). Alvarez-Silva and Borojevic identified heparan sulfate secreted by cells isolated from murine hepatic schistosomal granulomas, which could interact with growth factors *in vitro*, such as G-CSF, and act like a myelopoietic stroma (31). However, our data showed the absence of the sulfated and acid glycoconjugates in regions of hematopoietic proliferation in the schistosomal liver, although it is present in medial zone of granuloma. This led us to suggest that the cells isolated by Alvarez-Silva and Borojevic were from medial zone of granuloma and the extramedullary hematopoiesis which happens in the peripheral zone of granuloma is independent of sulfated and acid glycoconjugates, at least by direct cell-matrix interactions.

MMP9 is a metalloproteinase involved in tissue remodeling through wide-spectrum extracellular matrix degradation, which can occur under pathological or physiological conditions (32). At 35 dpi, MMP9 was expressed by neutrophils inside sinusoids or in areas of necrosis, indicating their participation in the initial histolysis in the liver caused by the arrival of the parasite eggs. Inside the granulomas, at 50 and 60 dpi, the MMP9-positive neutrophils were located along the peripheral zone. In addition to acting in the remodeling of the extracellular matrix, Kawai et al. showed that this molecule can act as chemoattractant of hematopoietic cells Lin^-^ in liver damage. The mechanism involves an expression enhancement of CXCR4 and its ligand, SDF-1, which is found in a concentration gradient towards the organ (33). Considering the liver damage caused by schistosomal infection, it is possible that MMP9 also can attract immature hematopoietic cells to cellular proliferation regions, especially in the periphery of granulomas.

In addition to extramedullary hematopoiesis, schistosomal infection may promotes angiogenesis in the liver, as previously reported (7, 8, 34). We confirmed the increase in the number of blood vessels in mature granulomas using the immunolabeling by CD31 molecules. Usually, angiogenesis in schistosomal liver can be associated to hypoxia caused by granulomatous and periportal fibrosis. The generation of vessels caused by hypoxia can be related to expression of HIF, which could stimulate the release of VEGF and promotes the proliferation of endothelial cells (35, 36). Also, it has already been demonstrated that SEA regulates positivity the expression of VEGF (37) and consequently promotes the endothelial proliferation and angiogenesis (38). Furthermore, we identified lymphatic vessels in the peripheral zone of mature granulomas, by immunofluorescence for LYVE-1. This hyaluronan receptor is mainly expressed in lymphatic vessels and can be involved in process of lymphangiogenesis (39, 40).

Although vWF is normally present by endothelial cells, there is a differential expression in different organs (41). Our data showed some blood vessels expressing vWF, mainly large vessels and sinusoids associated to hematopoietic cells. Previous studies have shown that the absence of vWF can lead to endothelial cell proliferation in two ways: 1) vWF participates in formation of Weibel-Palade bodies, which store Angiopoietin-2 (Ang-2). So, in the absence of vWF, Ang-2 links to Tie-2 and act synergically to VEGFR2, promoting angiogenesis; 2) the absence of vWF decrease the expression of αvβ3 and it leads to increase the quantity of membrane VEGFR2, promoting angiogenesis (41–43). Based on this data, we suggest that angiogenesis possibly occur manly from some sinusoids which do not express vWF.

In addition to blood vessels, vWF is classically described as a molecule which also identifies cells of the megakaryocytic lineage (44). In addition to the increase of proliferating myeloid cells, the murine schistosomal liver also showed an apparent increase in cells of the megakaryocytic lineage. More than one megakaryocyte was frequently seen together, leading us to consider the possibility of local proliferation. To explain this phenomenon, we suggest two possibilities: 1) Chemoattraction of megakaryocytic progenitors to the schistosomal liver, which find favorable microenvironment to proliferate and differentiate; 2) Attraction of undifferentiated HSC or HSPC, which can differentiate both in other hematopoietic cells and directly in megakaryocytic progenitors or megakaryocytes. The second hypothesis is based on the holocracy theory of hematopoiesis, in which HSC could be capable of differentiate into restrict hematopoietic progenitors, such as Common myeloid progenitor (CMP), Megakaryocyte Erythroid Progenitor (MEP) and Megakaryocyte Progenitor (MkP) (45, 46). Some LT-HSC (Long Term Hematopoietic Stem Cell) which express vWF were stimulated with thrombopoietin and could be capable of maintaining the production of megakaryocytes and platelets for long term (47).

Considering the possibility of the arrival of immature hematopoietic cells to the liver, analyzes were performed to identify Sca-1 expressing cells. This molecule is used as a marker of immature hematopoietic cells and can be present in LT-HSC, ST-HSC (Short Term Hematopoietic Stem Cell) and MMP (Multipotent Progenitor) (48). In this work, we identified hematopoietic cells expressing Sca-1 in mature granulomas, indicating the presence of more immature cells than those previously observed by Lenzi et al. (9), who, by brightfield microscopy, identified promyelocytes and metamyelocytes (15). The expression of Sca-1 was also observed in some blood vessels, which is already described for mice. According to Luna et al., (49), the expression of Sca-1 by endothelial cells can support the hypothesis of a common progenitor to hematopoietic and endothelial cells (49).

The schistosomal liver is an environment conducive to the occurrence of angiogenesis and hematopoiesis. The possibility of common progenitors between endothelial and hematopoietic cells is based on the characteristics of the hemogenic endothelium, which occurs during the embryonic life of mammals. Cells derived from this structure are part of the definitive hematopoietic wave, although some of these cells have already been found in adult individuals (50–52). These data, together with the characteristics shared with the hematopoietic fetal liver, lead us to suggest that schistosomiasis is a disease capable of activating a context that contribute to the establishment of a hepatic environment similar to the one found during embryonic development.

The immunolabeling of Fall-3 also was chosen to identify immature hematopoietic cells. In addition to identifying HSC and HSPC, Fall3 is present both in ly6^a^ and ly6^b^ cells which suggests that this marker is also capable of identifying more differentiated cells (53). The morphological data generated by the Fall-3 labeling provide us with important information about the phenotypic characterization of cells present in regions of proliferation. It was possible to detect labelled large cells with loose chromatin and protruding nucleolus, that are characteristics commonly found in immature cells. These morphological data reinforce the hypothesis of the arrival of progenitors to the liver that are very immature.

The differences in pattern of distribution of cells and extracellular matrix in zones of schistosomal granuloma can lead us to consider that the structure is analogous to an ecosystem, as suggested by Malta et. Al., 2021 (54). In this context, hematopoietic cells would be occupying a specific niche in the peripheral zone of granuloma, which provide conditions of survival and maintenance of these cells interacting with each other and with cellular matrix elements. The idea of niche in ecological context of granuloma and in production of hematopoietic cells in bone marrow and in the embryological development presents some similarities, as we observed here.

From a physiological perspective, extramedullary hematopoiesis, both perivascular and perigranulomatous, increases the system’s ability to produce hematopoietic cells in a context in which the bone marrow is already highly activated and with intense hematopoietic activity. This is possible because, at the same time that the bone marrow is exporting immature cells, either because of the hyperplasia itself, or because of the possible direct mobilization of hematopoietic progenitors, the assembly of hepatic microenvironments favorable to its homing and proliferation is taking place. In addition to a coincidence of events, perhaps this may have some evolutionary importance in the co-evolution of Schistosoma and its mammalian host.

In summary, the present work describes new aspects about hepatic extramedullary hematopoiesis that occurs during murine schistosomal infection. We detailed the characteristics of the perigranulomatous and perivascular niches as microenvironments conducive to the proliferation of hematopoietic cells, in addition to shedding light on the possibility of integrating the processes of hematopoiesis and hepatic angiogenesis triggered by the disease. Also, our data suggests the possibility of direct mobilization of immature hematopoietic cells from bone marrow to both hepatic sites, induced by SEA, which should be a topic of further investigation.

## Data availability statement

The raw data supporting the conclusions of this article will be made available by the authors, without undue reservation.

## Ethics statement

The animal study was reviewed and approved by Institutional Ethics Committee for Animal Research of the Oswaldo Cruz Institute (CEUA IOC, license: L-2/13).

## Author contributions

JF, MT, BD, and MP-M contributed to conception and design of the study. JF, MT, GK, and BD collected samples and executed histology techniques. JF, MT, and MP-M made immunofluorescence analysis. JF and MP-M wrote the manuscript. All authors contributed to manuscript revision, read, and approved the submitted version.

## Funding

This work was supported by the Brazilian National Research Council (CNPq INCT-NIM #465489/2014-1 and PQ 313520-2018-6) and the Oswaldo Cruz Institute/Fiocruz (intitutional funding). JF and GK were fellows from the Coordination for the Improvement of Higher Education Personnel (CAPES). The funders had no role in study design, data collection/analysis, decision to publish, or preparation of the manuscript.

## Acknowledgments

The authors would like to thank Ester Maria Mota for scientific discussions, Filomena de Fátima Cruz, Iolanda Deolinda de Souza and Monique Lima for the infection of mice with *Schistosoma mansoni* cercariae; João Paulo Rodrigues, Luciana Silva Souza, Luzia Fátima Caputo, Rakel Vieira de Souza and Thalita Paschoal for histotechnology assistance; and also Giulia Caminha and Pedro Paulo de Abreu Manso for support concerning confocal microscopy.

## Conflict of interest

The authors declare that the research was conducted in the absence of any commercial or financial relationships that could be construed as a potential conflict of interest.

## Publisher’s note

All claims expressed in this article are solely those of the authors and do not necessarily represent those of their affiliated organizations, or those of the publisher, the editors and the reviewers. Any product that may be evaluated in this article, or claim that may be made by its manufacturer, is not guaranteed or endorsed by the publisher.
